# Supplementary material for: Beta-Lactam vs. Fluoroquinolone Monotherapy for Pseudomonas aeruginosa Infection: A Systematic Review and Meta-Analysis
Source: Antibiotics (Basel). 2021 Dec 3;10(12):1483. doi: 10.3390/antibiotics10121483 (PMC8698261; doi:10.3390/antibiotics10121483)
Supplement: Supplementary file 1 [file antibiotics-10-01483-s001.zip › File S1-Medline Search Strategy.pdf]

Thursday, April 18, 2019 1:46:08 PM

#	Query	Limiters/Expanders	Last Run Via	Results
S18	s11 NOT s16	Limiters - English Language; Human<br>Search modes - Boolean/Phrase	Interface - EBSCOhost Research Databases<br>Search Screen - Advanced Search<br>Database - MEDLINE Complete	171
S17	s11 NOT s16	Limiters - English Language<br>Search modes - Boolean/Phrase	Interface - EBSCOhost Research Databases<br>Search Screen - Advanced Search<br>Database - MEDLINE Complete	225
S16	S11 AND S14	Limiters - English Language<br>Search modes - Boolean/Phrase	Interface - EBSCOhost Research Databases<br>Search Screen - Advanced Search<br>Database - MEDLINE Complete	62
S15	S11 AND S14	Search modes - Boolean/Phrase	Interface - EBSCOhost Research Databases<br>Search Screen - Advanced Search<br>Database - MEDLINE Complete	81
S14	S12 OR S13	Search modes - Boolean/Phrase	Interface - EBSCOhost Research Databases<br>Search Screen - Advanced Search<br>Database - MEDLINE Complete	120,095
S13	iatrogen* OR nosocomial* OR hospital N2 acqui* OR	Search modes - Boolean/Phrase	Interface - EBSCOhost Research Databases<br>Search Screen - Advanced	119,045

	healthcare N2 acqui* OR healthcare N2 associat* OR cross N2 infect*		Search<br>Database - MEDLINE Complete	
S12	(MH "Iatrogenic Disease+") OR (MH "Infectious Disease Transmission, Professional-to-Patient")	Search modes - Boolean/Phrase	Interface - EBSCOhost Research Databases<br>Search Screen - Advanced Search<br>Database - MEDLINE Complete	70,808
S11	S7 AND S10	Search modes - Boolean/Phrase	Interface - EBSCOhost Research Databases<br>Search Screen - Advanced Search<br>Database - MEDLINE Complete	332
S10	S8 OR S9	Search modes - Boolean/Phrase	Interface - EBSCOhost Research Databases<br>Search Screen - Advanced Search<br>Database - MEDLINE Complete	73,460
S9	pseudomonas N2 infect* OR aeruginosa	Search modes - Boolean/Phrase	Interface - EBSCOhost Research Databases<br>Search Screen - Advanced Search<br>Database - MEDLINE Complete	73,460
S8	(MH "Pseudomonas Infections") OR (MH "Pseudomonas aeruginosa")	Search modes - Boolean/Phrase	Interface - EBSCOhost Research Databases<br>Search Screen - Advanced Search<br>Database - MEDLINE Complete	48,259
S7	S5 AND S6	Search modes - Boolean/Phrase	Interface - EBSCOhost Research Databases<br>Search Screen - Advanced Search<br>Database - MEDLINE Complete	1,850

S6	monotherap* or (mono N1 therap*)	Search modes - Boolean/Phrase	Interface - EBSCOhost Research Databases<br>Search Screen - Advanced Search<br>Database - MEDLINE Complete	46,355
S5	S1 OR S2 OR S3 OR S4	Search modes - Boolean/Phrase	Interface - EBSCOhost Research Databases<br>Search Screen - Advanced Search<br>Database - MEDLINE Complete	180,992
S4	(MH "beta-Lactams+")	Search modes - Boolean/Phrase	Interface - EBSCOhost Research Databases<br>Search Screen - Advanced Search<br>Database - MEDLINE Complete	124,347
S3	fluoroquinolone? OR ciprofloxacin OR levofloxacin	Search modes - Boolean/Phrase	Interface - EBSCOhost Research Databases<br>Search Screen - Advanced Search<br>Database - MEDLINE Complete	45,996
S2	(beta N1 lactam?) OR aztreonam OR cefepime OR ceftazidime OR imipenem OR meropenem OR doripenem OR piperacillin OR tazobactam OR piperacillin/tazobactam	Search modes - Boolean/Phrase	Interface - EBSCOhost Research Databases<br>Search Screen - Advanced Search<br>Database - MEDLINE Complete	42,961
S1	(MH "Fluoroquinolones+")	Search modes - Boolean/Phrase	Interface - EBSCOhost Research Databases<br>Search Screen - Advanced Search<br>Database - MEDLINE Complete	31,084
