# Supplementary material for: Beta-Lactam vs. Fluoroquinolone Monotherapy for Pseudomonas aeruginosa Infection: A Systematic Review and Meta-Analysis
Source: Antibiotics (Basel). 2021 Dec 3;10(12):1483. doi: 10.3390/antibiotics10121483 (PMC8698261; doi:10.3390/antibiotics10121483)
Supplement: Supplementary file 1 [file antibiotics-10-01483-s001.zip › File S2-Quality Assessments.pdf]

Study Title: Treatment of severe pneumonia in hospitalized patients: results of a multicenter, randomized, double-blind trial comparing intravenous ciprofloxacin with imipenem-cilastatin
Authors: Fink, et al
Year: 1994

Quality Assessment of Controlled Intervention Studies

Item	Question	Yes	No	Other	Location
1	Was the study described as randomized, a randomized trial, a randomized clinical trial, or an RCT?	X			Methods ¶1
2	Was the method of randomization adequate (i.e., use of randomly generated assignment)?	X			Methods ¶3
3	Was the treatment allocation concealed (so that assignments could not be predicted)?	X			Methods ¶14
4	Were study participants and providers blinded to treatment group assignment?	X			Methods ¶1
5	Were the people assessing the outcomes blinded to the participants' group assignments?	X			Methods ¶11,14
6	Were the groups similar at baseline on important characteristics that could affect outcomes (e.g., demographics, risk factors, co-morbid conditions)?	X			Results ¶2, table 2
7	Was the overall drop-out rate from the study at endpoint 20% or lower of the number allocated to treatment?		X		Results ¶2
8	Was the differential drop-out rate (between treatment groups) at endpoint 15 percentage points or lower?	X			Results ¶2
9	Was there high adherence to the intervention protocols for each treatment group?	X			Results ¶4,5
10	Were other interventions avoided or similar in the groups (e.g., similar background treatments)?		X		Results ¶4,5
11	Were outcomes assessed using valid and reliable measures, implemented consistently across all study participants?			CD	
12	Did the authors report that the sample size was sufficiently large to be able to detect a difference in the main outcome between groups with at least 80% power?		X		
13	Were outcomes reported or subgroups analyzed prespecified (i.e., identified before analyses were conducted)?	X			Discussion ¶1, Methods ¶10,11
14	Were all randomized participants analyzed in the group to which they were originally assigned, i.e., did they use an intention-to-treat analysis?	X			Methods ¶14

Quality Rating (Good, Fair, Poor)

Rater	Rating	Initials	Comments
1	Good	ECR	Outcome: microbiological eradication<br>Issues: use of other drugs (Results ¶5, Methods ¶7), polymicrobial infx (approximately ½ of all participants, Results ¶9), unblinded study pharmacist (Methods ¶5)

2	Good	RW	Missing power

Study Title: Factors associated with improved outcome of PA bacteremia in a Finnish university hospital
Authors: Kuikka, et al
Year: 1998

Quality Assessment for Observational Cohort and Cross-Sectional Studies

Item	Question	Yes	No	Other	Location
1	Was the research question or objective in this paper clearly stated?	X			Intro ¶1,2
2	Was the study population clearly specified and defined?	X			Methods ¶1
3	Was the participation rate of eligible persons at least 50%?			N/A	Methods
4	Were all the subjects selected or recruited from the same or similar populations (including the same time period)? Were inclusion and exclusion criteria for being in the study prespecified and applied uniformly to all participants?		X		Methods ¶1
5	Was a sample size justification, power description, or variance and effect estimates provided?		X		
6	For the analyses in this paper, were the exposure(s) of interest measured prior to the outcome(s) being measured?	X			Methods
7	Was the timeframe sufficient so that one could reasonably expect to see an association between exposure and outcome if it existed?	X			Methods
8	For exposures that can vary in amount or level, did the study examine different levels of the exposure as related to the outcome (e.g., categories of exposure, or exposure measured as continuous variable)?		X		
9	Were the exposure measures (independent variables) clearly defined, valid, reliable, and implemented consistently across all study participants?	X			Methods
10	Was the exposure(s) assessed more than once over time?	X			Methods
11	Were the outcome measures (dependent variables) clearly defined, valid, reliable, and implemented consistently across all study participants?		X		
12	Were the outcome assessors blinded to the exposure status of participants?			NA	
13	Was loss to follow-up after baseline 20% or less?			NA	
14	Were key potential confounding variables measured and adjusted statistically for their impact on the relationship between exposure(s) and outcome(s)?		X		

Quality Rating (Good, Fair, Poor)

Rater	Rating	Initials	Comments

1	Fair	ECR	Outcome: mortality<br>PA-specific study, 111 on appropriate definitive abx vs 134 total eligible cases
2	Fair	RW	Treatment was secondary prognostic factor of death and it was one of many

Study Title: Clinafloxacin versus Piperacillin-tazobactam in treatment of patients with severe skin and soft tissue infections
Authors: Siami, et al
Year: 2001

Quality Assessment of Controlled Intervention Studies

Item	Question	Yes	No	Other	Location
1	Was the study described as randomized, a randomized trial, a randomized clinical trial, or an RCT?	X			Methods ¶1
2	Was the method of randomization adequate (i.e., use of randomly generated assignment)?			CD/NR	
3	Was the treatment allocation concealed (so that assignments could not be predicted)?			CD/NR	
4	Were study participants and providers blinded to treatment group assignment?		X		
5	Were the people assessing the outcomes blinded to the participants' group assignments?	X			Methods ¶8
6	Were the groups similar at baseline on important characteristics that could affect outcomes (e.g., demographics, risk factors, co-morbid conditions)?	X			Results ¶1, Table 1
7	Was the overall drop-out rate from the study at endpoint 20% or lower of the number allocated to treatment?		X		Results ¶2
8	Was the differential drop-out rate (between treatment groups) at endpoint 15 percentage points or lower?	X			Results ¶2
9	Was there high adherence to the intervention protocols for each treatment group?		X		
10	Were other interventions avoided or similar in the groups (e.g., similar background treatments)?		X		
11	Were outcomes assessed using valid and reliable measures, implemented consistently across all study participants?			CD	
12	Did the authors report that the sample size was sufficiently large to be able to detect a difference in the main outcome between groups with at least 80% power?	X			Methods ¶15
13	Were outcomes reported or subgroups analyzed prespecified (i.e., identified before analyses were conducted)?	X			Efficacy and safety analyses paragraph
14	Were all randomized participants analyzed in the group to which they were originally assigned, i.e., did they use an intention-to-treat analysis?		X		

Quality Rating (Good, Fair, Poor)

Rater	Rating	Initials	Comments
1	Poor	ECR	Outcome: microbiological response<br>Issues: polymicrobial infx (Results ¶5), only 7 true PA monoinfx (Results ¶5), option for vanco (Methods ¶2), also receipt of nonallowed antibacterial agents possible (Methods ¶9,12), not all in-patients (abstract), completers/per protocol analysis for clinical cure and microbiological eradication (Methods ¶12), inclusion of pts who took another antimicrobial were included in microbioloical analysis (Methods ¶12)<br><br>Significant potential for bias exists due to non-ITT analysis of main outcomes, unknown rates of patients using nonprotocol drugs, the option for vancomycin in the piptazo group (even if it is to account for gram + coverage conveyed by clinafloxacin), and single-blind design.
2	Poor	RW	

Study Title: Antibiotic Therapy and Clinical Outcomes of PA Bacteremia
Authors: Tan, et al
Year: 2014

Quality Assessment for Observational Cohort and Cross-Sectional Studies

Item	Question	Yes	No	Other	Location
1	Was the research question or objective in this paper clearly stated?	X			Intro ¶2,3,4
2	Was the study population clearly specified and defined?	X			Methods ¶1
3	Was the participation rate of eligible persons at least 50%?			NA	
4	Were all the subjects selected or recruited from the same or similar populations (including the same time period)? Were inclusion and exclusion criteria for being in the study prespecified and applied uniformly to all participants?	X			Methods ¶1
5	Was a sample size justification, power description, or variance and effect estimates provided?		X		
6	For the analyses in this paper, were the exposure(s) of interest measured prior to the outcome(s) being measured?	X			Methods
7	Was the timeframe sufficient so that one could reasonably expect to see an association between exposure and outcome if it existed?			CD	Methods
8	For exposures that can vary in amount or level, did the study examine different levels of the exposure as related to the outcome (e.g., categories of exposure, or exposure measured as continuous variable)?		X		Methods
9	Were the exposure measures (independent variables) clearly defined, valid, reliable, and implemented consistently across all study participants?	X*			Methods
10	Was the exposure(s) assessed more than once over time?	X			Methods

11	Were the outcome measures (dependent variables) clearly defined, valid, reliable, and implemented consistently across all study participants?		X		
12	Were the outcome assessors blinded to the exposure status of participants?			NA	
13	Was loss to follow-up after baseline 20% or less?			NA	
14	Were key potential confounding variables measured and adjusted statistically for their impact on the relationship between exposure(s) and outcome(s)?		X		

Quality Rating (Good, Fair, Poor)

Rater	Rating	Initials	Comments
1	Fair	ECR	ComboTx pts had more CVD, DM, vascular access as infx source<br>Issues:<br>Polymicrobial bacteremia in 19 cases; (confounding variables?) significantly more pts with CVD, cancer, HIV/AIDS, higher SAPS II score of 19 deaths → adjusted for in multivariable analysis; small sample size
2	Fair	RW	Could've done more years of data to do a strong power analysis

*3/4 criteria met, validity is CD based on definition of definitive therapy being 2 days and approximately 40% of pts receiving active empirical therapy.

Study Title: Treatment of severe nosocomial pneumonia: a prospective randomized comparison of intravenous ciprofloxacin and imipenem/cilastatin
Authors: Torres, et al
Year: 2000

Quality Assessment of Controlled Intervention Studies

Item	Question	Yes	No	Other	Location
1	Was the study described as randomized, a randomized trial, a randomized clinical trial, or an RCT?	X			Methods ¶1
2	Was the method of randomization adequate (i.e., use of randomly generated assignment)?			NR/CD	
3	Was the treatment allocation concealed (so that assignments could not be predicted)?	X			Methods ¶6
4	Were study participants and providers blinded to treatment group assignment?		X		Methods ¶1
5	Were the people assessing the outcomes blinded to the participants' group assignments?			NR	
6	Were the groups similar at baseline on important characteristics that could affect outcomes (e.g., demographics, risk factors, co-morbid conditions)?	X			Table 1
7	Was the overall drop-out rate from the study at endpoint 20% or lower of the number allocated to treatment?		X		Results ¶1, Figure 1

8	Was the differential drop-out rate (between treatment groups) at endpoint 15 percentage points or lower?	X			Results ¶1, Figure 1
9	Was there high adherence to the intervention protocols for each treatment group?			CD	
10	Were other interventions avoided or similar in the groups (e.g., similar background treatments)?			CD	
11	Were outcomes assessed using valid and reliable measures, implemented consistently across all study participants?			CD	
12	Did the authors report that the sample size was sufficiently large to be able to detect a difference in the main outcome between groups with at least 80% power?		X		
13	Were outcomes reported or subgroups analyzed prespecified (i.e., identified before analyses were conducted)?	X			Methods
14	Were all randomized participants analyzed in the group to which they were originally assigned, i.e., did they use an intention-to-treat analysis?	X			Methods ¶14

Quality Rating (Good, Fair, Poor)

Rater	Rating	Initials	Comments
1	Fair	ECR	Outcomes: clinical and microbiologic response<br>Issues: polymicrobial infx (Methods ¶8), allowed for antimicrobial agents if started more than five days before the study (Methods ¶10)<br><br>Although there are issues with not reporting information needed to adequately answer some of the questions, the authors openly talk about disclosing possible biases and utilize an ITT analysis.
2	Fair	RW	

Study Title: Is fluoroquinolone monotherapy a useful alternative treatment for PA bacteremia?
Authors: Wu, et al
Year: 2018

Quality Assessment for Observational Cohort and Cross-Sectional Studies

Item	Question	Yes	No	Other	Location
1	Was the research question or objective in this paper clearly stated?	X			Introduction ¶2,3
2	Was the study population clearly specified and defined?	X			Methods ¶1,2
3	Was the participation rate of eligible persons at least 50%?			NA	Results ¶1
4	Were all the subjects selected or recruited from the same or similar populations (including the same time period)? Were inclusion and exclusion criteria for being in the study prespecified and applied uniformly to all participants?	X			Methods ¶1,2

5	Was a sample size justification, power description, or variance and effect estimates provided?		X		
6	For the analyses in this paper, were the exposure(s) of interest measured prior to the outcome(s) being measured?	X			Methods
7	Was the timeframe sufficient so that one could reasonably expect to see an association between exposure and outcome if it existed?	X			Methods
8	For exposures that can vary in amount or level, did the study examine different levels of the exposure as related to the outcome (e.g., categories of exposure, or exposure measured as continuous variable)?		X		Methods definitions
9	Were the exposure measures (independent variables) clearly defined, valid, reliable, and implemented consistently across all study participants?	X			Methods
10	Was the exposure(s) assessed more than once over time?	X			Methods
11	Were the outcome measures (dependent variables) clearly defined, valid, reliable, and implemented consistently across all study participants?	X			Methods definitions
12	Were the outcome assessors blinded to the exposure status of participants?			NA	
13	Was loss to follow-up after baseline 20% or less?			NA	
14	Were key potential confounding variables measured and adjusted statistically for their impact on the relationship between exposure(s) and outcome(s)?	X			Methods statistical analysis ¶1, table 5

Quality Rating (Good, Fair, Poor)

Rater	Rating	Initials	Comments
1	Good	ECR	Outcome: mortality<br>Excluded polymicrobial infx; pts could switch to po FQ, community acquired infx possible.<br>Possible issue with pts could be changed to another active antipseudomonal but were excluded and separate analysis was done, Results ¶4 discussion ¶4.
2	Good	RW	
