# Supplementary material for: Beta-Lactam vs. Fluoroquinolone Monotherapy for Pseudomonas aeruginosa Infection: A Systematic Review and Meta-Analysis
Source: Antibiotics (Basel). 2021 Dec 3;10(12):1483. doi: 10.3390/antibiotics10121483 (PMC8698261; doi:10.3390/antibiotics10121483)
Supplement: Supplementary file 1 [file antibiotics-10-01483-s001.zip › File S3-Scopus Search Strategy.pdf]

Scopus

Advanced search

Documents Authors Affiliations Advanced Search tips ?

Enter query string

(((TITLE-ABS-KEY(fluoroquinolone? OR ciprofloxacin OR levofloxacin) OR TITLE-ABS-KEY((beta W/1 lactam?) OR aztreonam OR cefepime OR ceftazidime OR imipenem OR meropenem OR doripenem OR piperacillin OR tazobactam OR piperacillin/tazobactam))) AND ((((TITLE-ABS-KEY (monotherap*) or TITLE-ABS-KEY(mono W/1 therap*))) AND (TITLE-ABS-KEY(pseudomonas W/2 infect*) OR TITLE-ABS-KEY (aeruginosa))) AND TITLE-ABS-KEY ((iatrogen* OR nosocomial* OR (hospital W/2 acqui*) OR (healthcare W/2 acqui*) OR (healthcare W/2 associat*) OR (cross W/2 infect*))))) AND (TITLE-ABS-KEY (human)) AND (LIMIT-TO (LANGUAGE,"English"))

Outline query Add Author name / Affiliation Clear form Search

ALL("Cognitive architectures") AND AUTHOR-NAME(smith)
TITLE-ABS-KEY(*somatic complaint wom?n) AND PUBYEAR AFT 1993
SRCTITLE(*field ornith*) AND VOLUME(75) AND ISSUE(1) AND PAGES(53-66)

Operators

AND
OR
AND NOT
PRE/
W/

Field codes ?

Textual Content
Affiliations
Authors
Biological Entities
Chemical Entities
Conferences
Document
Editors
Funding
Keywords
Publication
References
Subject Areas

Search history

Combine queries... *e.g. #1 AND NOT #3*

11	(((TITLE-ABS-KEY (fluoroquinolone? OR ciprofloxacin OR levofloxacin) OR TITLE-ABS-KEY ((beta W/1 lactam?) OR aztreonam OR cefepime OR ceftazidime OR imipenem OR meropenem OR doripenem OR piperacillin OR tazobactam OR piperacillin/tazobactam))) AND ((((TITLE-ABS-KEY (monotherap*) OR TITLE-ABS-KEY (mono W/1 therap*))) AND (TITLE-ABS-KEY (pseudomonas W/2 infect* ... View More	164 document results
10	((TITLE-ABS-KEY (fluoroquinolone? OR ciprofloxacin OR levofloxacin) OR TITLE-ABS-KEY ((beta W/1 lactam?) OR aztreonam OR cefepime OR ceftazidime OR imipenem OR meropenem OR doripenem OR piperacillin OR tazobactam OR piperacillin/tazobactam))) AND ((((TITLE-ABS-KEY (monotherap*) OR TITLE-ABS-KEY (mono W/1 therap*))) AND (TITLE-ABS-KEY (pseudomonas W/2 infect* ... View More	166 document results
9	((TITLE-ABS-KEY (fluoroquinolone? OR ciprofloxacin OR levofloxacin) OR TITLE-ABS-KEY ((beta W/1 lactam?) OR aztreonam OR cefepime OR ceftazidime OR imipenem OR meropenem OR doripenem OR piperacillin OR tazobactam OR piperacillin/tazobactam))) AND ((((TITLE-ABS-KEY (monotherap*) OR TITLE-ABS-KEY (mono W/1 therap*))) AND (TITLE-ABS-KEY (pseudomonas W/2 infect* ... View More	336 document results
8	((TITLE-ABS-KEY (fluoroquinolone? OR ciprofloxacin OR levofloxacin) OR TITLE-ABS-KEY ((beta W/1 lactam?) OR aztreonam OR cefepime OR ceftazidime OR imipenem OR meropenem OR doripenem OR piperacillin OR tazobactam OR piperacillin/tazobactam))) AND ((((TITLE-ABS-KEY (monotherap*) OR TITLE-ABS-KEY (mono W/1 therap*))) AND ((pseudomonas W/2 infect*) OR aeruginosa ... View More	528 document results

7	((TITLE-ABS-KEY (fluoroquinolone? OR ciprofloxacin OR levofloxacin) OR TITLE-ABS-KEY ((beta W/1 lactam?) OR aztreonam OR cefepime OR ceftazidime OR imipenem OR meropenem OR doripenem OR piperacillin OR tazobactam OR piperacillin/tazobactam))) AND ((((title-abs-key AND monotherap* OR TITLE-ABS-KEY (mono W/1 therap*))) AND ((pseudomonas W/2 infect*) OR aeruginosa)) AND View More	0 document results	

Showing 5 most recent searches | View all 11

^ Top of page

Help improve Scopu

About Scopus

What is Scopus

Content coverage

Scopus blog

Scopus API

Privacy matters

Language

日本語に切り替える

切换到简体中文

切換到繁體中文

Русский язык

Customer Service

Help

Contact us

ELSEVIER

Terms and conditions ↗ Privacy policy ↗

Copyright © 2019 Elsevier B.V ↗. All rights reserved. Scopus® is a registered trademark of Elsevier B.V.

We use cookies to help provide and enhance our service and tailor content. By continuing, you agree to the use of cookies.

RELX Grou
